# Epilepsy in Onchocerciasis Endemic Areas: Systematic Review and Meta-analysis of Population-Based Surveys

**DOI:** 10.1371/journal.pntd.0000461

**Published:** 2009-06-16

**Authors:** Sébastien D. S. Pion, Christoph Kaiser, Fernand Boutros-Toni, Amandine Cournil, Melanie M. Taylor, Stefanie E. O. Meredith, Ansgar Stufe, Ione Bertocchi, Walter Kipp, Pierre-Marie Preux, Michel Boussinesq

**Affiliations:** 1 Unité Mixte de Recherche 145, Institut de Recherche pour le Développement (IRD) and University of Montpellier 1, Montpellier, France; 2 Basic Health Services Kabarole & Bundbugyo Districts, Fort Portal, Uganda; 3 Institut d'Epidémiologie Neurologique et de Neurologie Tropicale (EA3174), Faculté de Médecine, Limoges, France; 4 Office of HIV, STD and Hepatitis C Services, Arizona Department of Health Services, Phoenix, Arizona, United States of America; 5 134 Chemin du Recredoz, Divonne les Bains, France; 6 Peramiho Mission Hospital, Peramiho, United Republic of Tanzania (URT); 7 Diocèse de Bouar, Bouar, Central African Republic (CAR); 8 Department of Public Health Sciences, University of Alberta, Edmonton, Alberta, Canada; The University of Nottingham, United Kingdom

## Abstract

**Objective:**

We sought to evaluate the relationship between onchocerciasis prevalence and that of epilepsy using available data collected at community level.

**Design:**

We conducted a systematic review and meta-regression of available data.

**Data Sources:**

Electronic and paper records on subject area ever produced up to February 2008.

**Review Methods:**

We searched for population-based studies reporting on the prevalence of epilepsy in communities for which onchocerciasis prevalence was available or could be estimated. Two authors independently assessed eligibility and study quality and extracted data. The estimation of point prevalence of onchocerciasis was standardized across studies using appropriate correction factors. Variation in epilepsy prevalence was then analyzed as a function of onchocerciasis endemicity using random-effect logistic models.

**Results:**

Eight studies from west (Benin and Nigeria), central (Cameroon and Central African Republic) and east Africa (Uganda, Tanzania and Burundi) met the criteria for inclusion and analysis. Ninety-one communities with a total population of 79,270 individuals screened for epilepsy were included in the analysis. The prevalence of epilepsy ranged from 0 to 8.7% whereas that of onchocerciasis ranged from 5.2 to 100%. Variation in epilepsy prevalence was consistent with a logistic function of onchocerciasis prevalence, with epilepsy prevalence being increased, on average, by 0.4% for each 10% increase in onchocerciasis prevalence.

**Conclusion:**

These results give further evidence that onchocerciasis is associated with epilepsy and that the disease burden of onchocerciasis might have to be re-estimated by taking into account this relationship.

## Author Summary

Epilepsy is particularly common in tropical areas. One main reason is that many endemic infections have neurological consequences. In addition, the medical, social and demographic burden of epilepsy remains substantial in these countries where it is often seen as a contagious condition and where the aetiology is often undetermined. For several decades, field researchers had reported some overlapping between the geographical distributions of epilepsy and onchocerciasis, a parasitic disease caused by the filarial worm *Onchocerca volvulus* which afflicts some 40 million persons worldwide. Here, we conducted a statistical analysis of all the data available on the relationship between the two conditions to determine whether the proportion of people suffering from epilepsy in a community could be related to the frequency of onchocerciasis. The combined results of the eight studies carried out in west, central and east Africa indicate a close epidemiological association between the two diseases. Should a causative relationship be demonstrated, onchocerciasis, which is known as “river blindness” because of its most serious sequela and the distribution of its vectors, could thus also be called “river epilepsy”. More research is needed to determine the mechanisms explaining this association and to assess the burden of onchocerciasis-associated epilepsy.

## Introduction

Recent surveys, based on rapid epidemiological mapping of onchocerciasis (REMO), have revealed that the number of people infected with *Onchocerca volvulus*, the causative agent of onchocerciasis, had been largely underestimated. According to the latest REMO results obtained as part of the African programme for onchocerciasis control (APOC), 37 million are estimated to be infected with *O. volvulus* in Africa [1]. Accordingly, the Disability Adjusted Life Years (DALYs) lost per year due to onchocerciasis is estimated to be 1.49 million. This number is threefold higher than the previous figure published 2 years earlier [2] but probably still underestimates the real burden of disease attributable to onchocerciasis. The current DALY estimates account for blindness, visual impairment and onchocercal itching due to skin disease, yet it does not account for significant excess mortality due to heavy infections with *O. volvulus* [3].

The excess mortality of sighted individuals with high microfilarial loads suggests the operation of insidious and systemic involvement, neurologic involvement having been proposed as a possible candidate [4]. In particular, a link between onchocerciasis and epilepsy has been suspected since the 1930s [5]. Population-wide surveys found significant correlations between prevalence of epilepsy and that of onchocerciasis in Uganda [6] and between prevalence of epilepsy and community microfilarial load (CMFL) of onchocerciasis in Cameroon [7]. However, case-control studies evaluating the association between epilepsy and onchocerciasis reached diverse conclusions: significant association was demonstrated in areas where onchocerciasis prevalence exceeded 60% [7],[8], whereas no significant relationship was found in areas with lower onchocerciasis prevalence [9],[10],[11].

The present work aims at testing the hypothesis that in communities with high onchocerciasis endemicity, the prevalence of epilepsy will clearly exceed that found in communities of low onchocerciasis endemicity. We conducted a literature review of epidemiological studies addressing the issue of the onchocerciasis-epilepsy relationship and performed a meta-regression analysis including available population-based data to quantify the influence of onchocerciasis endemicity on the prevalence of epilepsy.

## Methods

### Data sources and search strategy

We searched PubMed and ISI Web of Knowledge up to February 2008, with neither past time limit nor language restriction, to identify population-based studies reporting on the prevalence of epilepsy in communities for which onchocerciasis prevalence was available or such prevalence could be estimated. We entered the following search terms and Boolean operators, for matches under any field: epilep* AND onchocerc*, epilep* AND *country* with *country* being iteratively one of the 11 OCP countries, 19 APOC countries, six OEPA countries, and Yemen. Titles and available abstracts were scanned for relevance, identifying papers requiring further consideration. Bibliographies of relevant articles were checked. Relevant theses and reports were also searched at the library of the *Institut d'Epidémiologie et de Neurologie Tropicale* (*IENT*, Limoges). The authors who initiated the study (SDSP & MB) also contacted researchers who were requested to provide information and/or data not included in the articles or other documents.

### Study selection

Studies were selected if both epilepsy prevalence and an indicator of onchocerciasis prevalence were available or such prevalence could be calculated. Inclusion criteria for subsequent analysis were set to incorporate studies: (1) carried out following a population-based design; (2) providing information on the methods used to diagnose epilepsy and onchocerciasis and (3) in which epilepsy prevalence was assessed in the general population, i.e. both in children and adults. Study communities with a sample of less than 10 subjects were discarded.

### Data extraction

Two authors (SDSP, MB) independently assessed eligibility and study quality, and extracted data. We recorded all basic parasitological and demographic information from each eligible study into a purpose-built database. The extracted data included demographic characteristics of the population examined (age range and sex), recruitment methods, and number and dates of previous community treatments with ivermectin. For onchocerciasis, specific information was recorded on methods used for parasitological examination. For epilepsy, details on sampling procedures, and definition of epilepsy were recorded.

### Quantitative data synthesis

To quantify the extent to which epilepsy prevalence is associated with onchocerciasis endemicity across the different studies, a meta-regression was performed. Epilepsy prevalence was defined as the outcome and onchocerciasis prevalence as the explanatory variable.

We used a logistic model to assess the relationship between prevalence of epilepsy and that of onchocerciasis. A random effect, capturing between-studies heterogeneity was subsequently incorporated into the model previously outlined. Significance of this effect was tested using the likelihood ratio test [12]. Logistic models were first estimated using the raw data collected during the review of literature. Because we had some reasons to think that some of the published data may have suffered from methodological bias, we developed some correction factors to standardize the data (see below). Logistic models (fixed-effect then random-effect models) were then estimated from the corrected data.

In addition, in order to test whether the relationship between prevalence of epilepsy and that of onchocerciasis was influenced by a specific study, each study was successively omitted from the whole database and the parameters re-estimated. Parameters of the different models were estimated using the non-linear regression procedure (NLMixed) provided in the SAS v8.1 software. This procedure provides Bayes empirical estimates of the study-specific random-effect [13].

### Standardisation of onchocerciasis and epilepsy prevalence

The prevalence of onchocerciasis was considered to have been measured by a standard procedure if it has been estimated in the general population (≥5 years old) using the onchocerciasis diagnostic method used by the Onchocerciasis Control Programme in West Africa (OCP): this entails taking a skin biopsy from each iliac crest, using a 2 mm Holth-type punch, and incubating it for 24 h in normal saline before searching for the presence of *O. volvulus* microfilariae [14]. Five of the eight studies included in the analysis [6],[8],[15],[16],[17] used a method for the diagnosis of infection with *O. volvulus* deviating from the OCP standard procedure. Table 1 summarises the types of deviation and the rationale for correction of the respective onchocerciasis prevalence values. Correction factors were determined using information from published comparative studies. Details on these standardisation processes are presented in the Supporting Information to this article.

**Table 1:** Methodological departures from the standard OCP procedure to assess onchocerciasis infection status and proposed procedures to minimize the effect of these deviations on onchocerciasis prevalence.

Reference (country)	Deviation from standard OCP method for evaluation of onchocerciasis prevalence	Rationale for correction/assumption	Method for correction (for reference see Supporting Information)
Druet-Cabanac et al. [15] (Central African Republic)	Prevalence assessed from nodule palpation	Relationship between prevalence based on nodule palpation and prevalence of microfilaridermia	Relationship estimated through non linear regression performed on data collected in the same onchocerciasis focus
Gbenou [16] (Benin)	Prevalence assessed from skin snips taken at either the scapula or at the iliac crests	Relationship between prevalence based on skin snips from the scapula and prevalence based on skin snips from the iliac crests	Relationship estimated through linear regression performed on published data
Gbenou [16](Benin)	Skin snips incubated for about 30 min	Relationship between the prevalence obtained at 30 min and at 24 h incubation time	Correction factor estimated from 16 villages with different endemicity levels
Kaiser et al. [6] (Uganda)	Prevalence assessed in individuals aged 10–23 years with a time of residency in the study area between 10–19 years	Relationship between prevalence in the general population and in the 10–19 years old population	Relationship estimated through linear regression performed on data collected in a similar onchocerciasis focus
Taylor et al. [17] (Tanzania)	Prevalence assessed from 5–7 mm skin snips taken at the lower anterior portion of one leg	Distribution of microfilariae in the skin of the shin is similar to that in the calf	Relationship estimated through linear regression performed on published data
Taylor et al. [17] (Tanzania)	Skin snips incubated for about 20 min	In terms of sensitivity, a large skin snip examined at 20 min similar to a standard snip read at 30 min, then a 30 min to 24 h correction is applied	Correction factor estimated from 16 villages with different endemicity levels
Newell et al. [8] (Burundi)	Prevalence assessed from scarification	Sensitivity to detect the presence of microfilariae with this method similar to the standard OCP method	No adjustment needed

In addition, three studies were carried out in areas where ivermectin treatment campaigns had been performed [6],[18],[19], which affected the measure of the prevalence of *O. volvulus* microfilaridermia. By using published studies on the effect of community treatment with ivermectin we developed a model to determine appropriate correction factors allowing us to estimate the pre-treatment prevalence according to the number of preceding treatment rounds (details given in Supporting Information).

In the present paper, we assumed that if epilepsy were associated with onchocerciasis, it might be due not only to the presence of parasites in the cerebral tissue, but also to cicatricial lesions persisting after the disappearance of the parasites after a treatment. This is the case for epilepsy induced by other infectious diseases (e.g. malaria [20]). Thus, we assumed that, in the present study, ivermectin treatments had little or no effect on the epilepsy of those patients who already suffered from this condition. Ivermectin treatments might decrease incidence of epilepsy but this effect would probably be perceptible after a number of years. This is supported by observations made by Kaiser et al. [21] who found that no major change in epilepsy incidence was observed over 4 years after the start of annual ivermectin mass treatment.

Four of the 8 studies included in the present analysis referred to the definition of epilepsy proposed by the International League Against Epilepsy (ILAE) in 1993 [22] of “two or more unprovoked seizures during the previous 2 years” whereas the remaining 4 studies used deviating definitions (Table 2). Characteristics of neuro-epidemiological methods as used in the different studies are presented in Table 2. Sampling procedures, methods for case identification and confirmatory examinations were not uniform and incompletely described in some studies. One study published by Kipp et al. [19] reported a particularly high epilepsy prevalence of 8% in the Kabarole district of western Uganda. However, for the diagnosis of epilepsy this study was confined to a rapid assessment of cases with suspected epilepsy without a more extensive confirmatory examination. A later survey conducted in the same onchocerciasis focus confirmed the diagnosis in only 61 (54%) out of 113 cases with suspected epilepsy [6]. If this ratio of confirmed over suspected epilepsy patients is applied to the survey of Kipp et al. [19], the crude epilepsy prevalence in the latter study should be revised from 8% to 4.3% (8%×0.54 = 4.3%).

**Table 2:** Synopsis of neuro-epidemiological methodology used in studies on epilepsy prevalence in areas endemic for onchocerciasis.

Reference (Country/No. of study sites)	Assessment of total population	Identification of possible cases	Confirmatory examination/staff qualification	Epilepsy definition
Gbenou [16] † (Benin/5)	Not specified	Door-to-door, random sample of households	Interview (non-standardized), medical examination/Neurologist	≥2 seizures*
Kaboré et al. [11] * (Burkina Faso/5)	Specific census	Door-to-door, exhaustive in population ≥15 year	Not specified/Neurologist	≥2 seizures
Newell et al. [8] (Burundi/1)	Not specified	Community leaders and staff of health centre	Not specified/Health agents supervised by medical doctor	≥4 grand mal seizures in preceding year if no AED‡
				≥1 grand mal seizure during preceding year if AED
Boussinesq et al. [7] (Cameroon/14)	National census	Lists from community leaders and systematic question on epilepsy during parasitological surveys	Interview (non-standardized)/Medical doctor	≥2 seizures during the previous 2 years
Druet-Cabanac et al. [15] (CAR/42)	Not specified	Door-to-door, exhaustive	Not specified/Health agents supervised by a medical doctor	Not specified
Dozie et al. [18] (Nigeria/13)	Specific census	Door-to-door, exhaustive (all households)	Interview (non-standardized), medical examination/Medical doctor trained in paediatric neurology	≥2 seizures within the previous 2 years
Taylor et al. [17] (Tanzania/3)	Not specified	Mobilisation of population (self-reporting)	Standardized questionnaire, medical examination/Medical doctor	≥2 seizures during the previous year
				≥1 seizure during the previous 5 years if AED
Kaiser et al. [6] † (Uganda/13)	Specific census	Door-to-door, exhaustive+Self-reporting patients with residency in study area	Standardized questionnaire, medical examination/Medical doctor trained in paediatric neurology	≥2 seizures within the previous 2 years
Kipp et al. [19] (Uganda/2)	Not specified	Door-to-door, random sample of households	Not specified/Medical doctor	≥2 grand mal seizures during the preceding year if no AED
				≥1 grand mal seizure during preceding 5 years if AED
Ovuga et al. [23] * (Uganda/1)	Not specified	Mobilisation of population (self-reporting)	Not specified/Neurologist	≥2 seizures during the previous year
				≥1 seizure during the previous 5 years if AED

*: Study excluded: see text.

**†:** Study site excluded:

a) Site 〈〈Masongora North〉〉 from Kaiser et al. [6], because only 8 individuals underwent parasitological examination.

b) Site 〈〈school〉〉 from Gbenou [16], because this comprised schoolchildren from different parts of the study area and consequently was not considered as a proper site.

**‡:** AED: individuals under anti-epileptic drug treatment. CAR: Central African Republic.

## Results

### Available data

**Figure 1:**
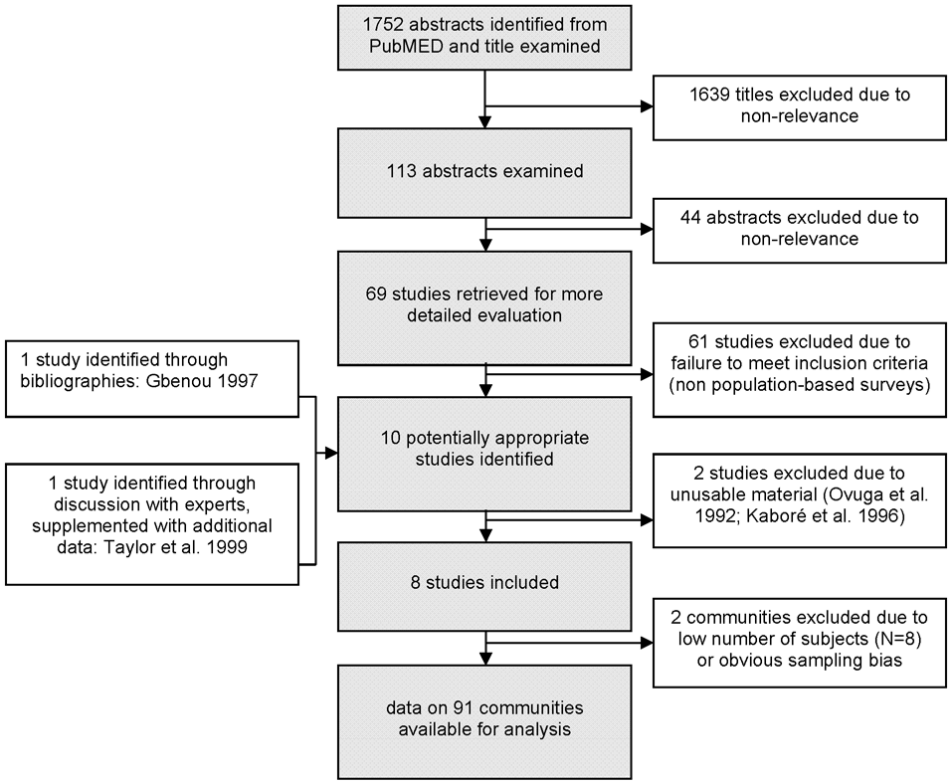
Flowchart summarizing the results of the search for papers on epilepsy potentially relating to onchocerciasis up to February 2008.

From the 1752 examined abstracts and research in the *IENT* library, ten different studies reporting population-based surveys on onchocerciasis and epilepsy prevalence were identified [6],[7],[8],[11],[15],[16],[17],[18],[19],[23] (Table 2). One study was excluded because it originated from an area where control measures had been carried out over 20 years within the Onchocerciasis Control Programme (OCP) and the overall prevalence had decreased to a low endemicity level at the time of the survey [10]. A second study focusing on the clinical description of a series of epilepsy patients in an onchocerciasis endemic area reported only an estimate of epilepsy prevalence in the study area and was also not considered [23]. A flowchart summary of the search is shown in Figure 1. Data meeting the inclusion criteria consisted of 91 communities from 8 distinct areas (2 in West Africa: Benin [16] and Nigeria [18]; 2 in Central Africa: Cameroon [7] and Central African Republic (CAR), [15] and 4 in East Africa: Uganda (2 sites) [6],[19], Tanzania [17] and Burundi [8],[24]). The different surveys took place between 1987 and 1997. Overall, 12 388 individuals were examined for *O. volvulus* infection and a total of 905 subjects with epilepsy were identified out of a screened population comprising 79 270 individuals (weighted average epilepsy prevalence: 1.14%). Epilepsy and onchocerciasis prevalences were assessed from the same population sample in two studies [16],[18] whereas in the six remaining studies [6],[7],[8],[15],[17],[19] this was done on different population samples in each community.

### Relationship between epilepsy prevalence and that of onchocerciasis

**Figure 2:**
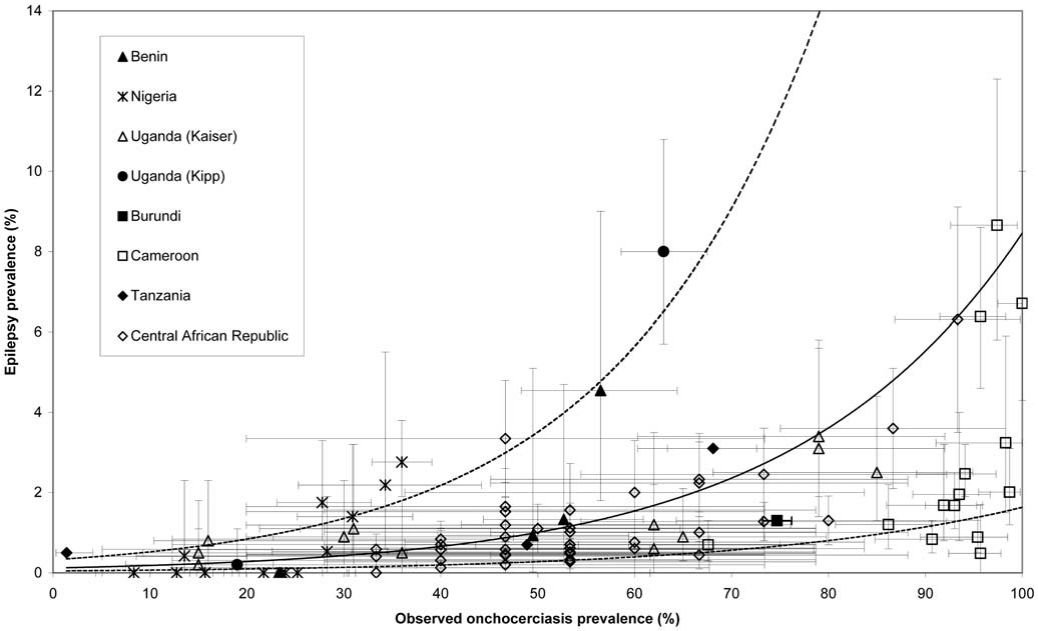
Epilepsy prevalence (as reported in the original studies) vs onchocerciasis prevalence. Error bars represent 95% exact confidence intervals. Solid line: predicted relationship estimated by random-effect logistic regression; dashed lines: 95% confidence interval of the model predictions.

**Figure 3:**
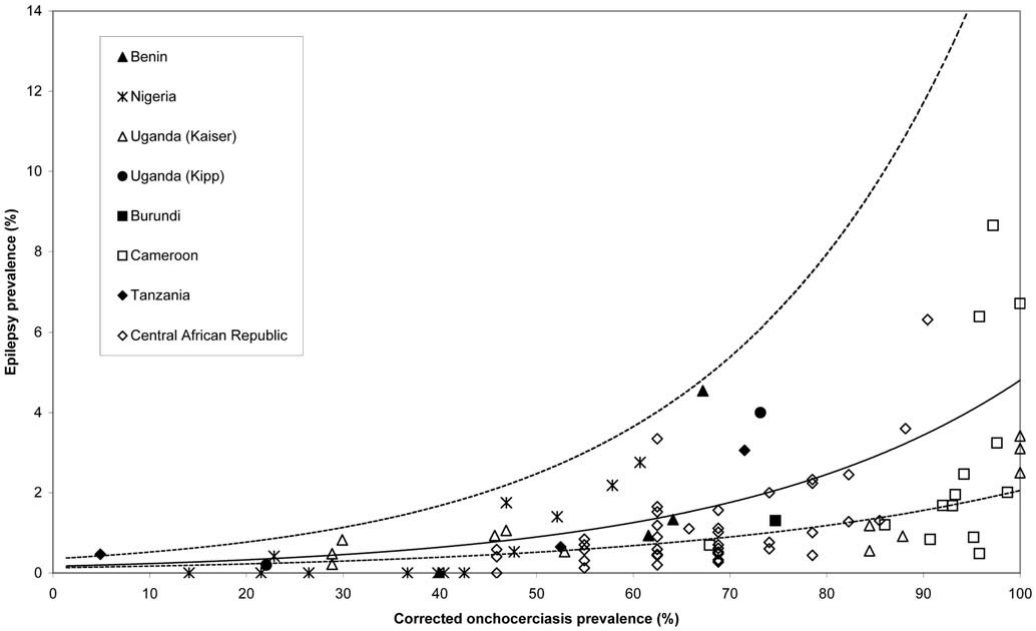
Epilepsy prevalence vs corrected onchocerciasis prevalence (see Supporting Information for details on correction factors). Solid line: predicted relationship estimated by random-effect logistic regression; dashed lines: 95% confidence interval of the model predictions.

Figure 2 represents epilepsy prevalence versus onchocerciasis prevalence, as reported in the documents retrieved during the review of literature, for the 91 study communities included in the analysis. Figure 3 represents the same data after applying the correction factors to minimize obvious biases. Epilepsy prevalence ranged from 0 to 8.7% and that of onchocerciasis from 1.4 to 100%. The fixed-effect logistic models fitted on observed and corrected data sets indicate a significant association (all parameters with p<0.0001) between onchocerciasis prevalence and epilepsy prevalence (Table 3). Inclusion of a random-effect resulted in a statistically significant improvement of the models (likelihood ratio tests p<0.0001 for both models, Table 3). The random-effect logistic model assessed on the corrected data provided slightly lower estimates than when assessed from observed data, with the respective corresponding odds-ratio: 1.042 (95%CI: 1.034–1.05) and 1.044 (95%CI: 1.036–1.052). These values indicate that, on average, epilepsy prevalence is increased by 0.4% for a 10% increase in onchocerciasis prevalence.

**Table 3:** Parameters estimated (and standard errors) from logistic regression of epilepsy (y) on onchocerciasis (x) prevalences.

Dataset	Model	*a* (S.E.)	*b* (S.E.)	U (S.E.)	-2LL
Observed data					
	Epilepsy prevalence vs. onchocerciasis prevalence estimates with fixed-effect model	−5.953 (0.136)	0.022 (0.002)	Fixed to 0	902.8
	Epilepsy prevalence vs. onchocerciasis prevalence estimates with random-effect model	−6.745 (0.386)	0.043 (0.003)	0.831 (0.435)	711.8
Corrected data					
	Epilepsy prevalence vs. onchocerciasis prevalence estimates with fixed-effect model	−6.589 (0.308)	0.029 (0.002)	Fixed to 0	761.2
	Epilepsy prevalence vs. onchocerciasis prevalence estimates with random-effect model	−7.048 (0.308)	0.041 (0.003)	0.308 (0.173)	690.2

The logistic model is expressed as 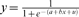, where u is the random effect parameter; -2LL: -2 Log likelihood.

However, as indicated by varying values of the random effect parameter *u* (Table 4), the influence of onchocerciasis on epilepsy differed between the studies. In particular, the random-effect model fitted on corrected data suggested that the influence of onchocerciasis on epilepsy was higher in the Nigeria study; conversely, it was lower in the Cameroon and Central African Republic studies (Table 4).

**Table 4:** Empirical Bayes estimates of the random effects due to inter-study heterogeneity from the regression models of epilepsy prevalence vs. onchocerciasis prevalence.

Study country	*u* (random effect)	Standard error	p-value
Benin [16]	0.455	0.336	0.22
Burundi [8]	−0.327	0.243	0.22
Cameroon [7]	−0.66	0.226	0.02
Central African Republic [15]	−0.538	0.211	0.04
Nigeria [18]	0.598	0.242	0.04
Tanzania [17]	0.427	0.234	0.11
Uganda [6]	−0.533	0.242	0.06
Uganda [19]	0.663	0.285	0.05

Sensitivity analysis showed that the significance of the association between onchocerciasis prevalence and that of epilepsy was not affected by omission of any of the studies (results not shown).

## Discussion

The present study was carried out in order to evaluate whether the epilepsy prevalence in communities living in *O. volvulus* endemic areas is related to the prevalence of onchocerciasis. All available community-based surveys on this subject were used. Throughout 91 communities distributed across 7 African countries, variation of epilepsy prevalence is associated with that of onchocerciasis prevalence.

A recent review took an approach different from ours to analyse epidemiological studies searching for a relationship between onchocerciasis and epilepsy [25]. This review examined studies from which it was possible to calculate the relative risk of epilepsy in patients being infected with *O. volvulus* compared to the risk of infection of inhabitants without epilepsy from the respective study area. Inconsistent results were observed for nine African studies providing sufficient data, with their respective relative risk calculated at a broad range from 0.84 to 6.80. The common relative risk of 1.21 (95% CL 0.99–1.47) for all studies was close to the threshold of significance, suggesting a probable but weak risk of epilepsy in onchocerciasis patients. Possibly, this review did not yield a more pronounced association because it included studies which were not originally designed as case-control studies. In areas of low endemicity, the possible effect of onchocerciasis on epilepsy in such an analysis will also be masked by the relatively higher proportion of epilepsy cases due to alternative aetiologies.

A major difference between the review of Druet-Cabanac et al. [25] and our analysis is that we introduced the quantitative dimension of *O. volvulus* infection by defining the onchocerciasis prevalence in a village as the risk factor. It has been demonstrated that onchocerciasis prevalence is closely related to the intensity of infection through a negative binomial relationship [26]. The mathematical properties of this relationship implies that the higher the prevalence, the higher the proportion of individuals with heavy microfilarial loads in the population. In a previous study [7], intensity of infection expressed as the microfilarial loads of patients with epilepsy was 2 to 3 fold higher than those of controls matched on age, sex, and village of residence. These results suggest that, as it has been found for ocular onchocercal pathology [27], intensity of infection with *O. volvulus* is a key factor in the induction of epilepsy. This would provide a further explanation as to why the results of the previous review [25] gave a weaker degree of significance to the association between epilepsy and onchocerciasis than those obtained in our study.

The random effect included in the modelling indicates that the influence of onchocerciasis on epilepsy varied between the studies. This heterogeneity can be due to either differences in the methodology used to assess the prevalence of onchocerciasis and/or that of epilepsy, or to true biological differences modulating the epilepsy/onchocerciasis association. In this respect, there is some indication that the onchocerciasis prevalence from the Nigerian study [18] may have been compromised by inadequate correction. We were not able to determine with precision the number of ivermectin treatments administered before the parasitologic survey, nor the time interval between the last ivermectin treatment and the survey. We assumed that the parasitological assessment had been conducted at least 10 months after the last treatment, which is a sufficient lapse of time for the majority of infected patients to present again with microfilariae in the skin. If the precise interval were shorter than our assumption, the true initial prevalence of onchocerciasis would have been higher than that used in our analysis. However, if one adds arbitrarily 20% to all prevalence rates in this survey, one obtains similar estimates for the random-effect model (*a* = −7.174 (S.E. = 0.301); *b* = 0.041 (0.003); -2Log Likelihood = 688.6), with the specific random-effect of this study being no more significant (P = 0.75).

In the Cameroonian study site, a 6-fold increase was found in the mortality rate among individuals with epilepsy compared to that in control individuals [28]. This significant mortality rate may have resulted in an underestimation of epilepsy prevalence when it was assessed through a cross-sectional survey. Should the mortality rate of people with epilepsy be lower in the Nigerian area, epilepsy prevalence in the latter would have been overestimated compared to those obtained in Cameroon. Lastly, we do not discard the possibility that other factors, such as *O. volvulus* strains with differing pathogenic potential [29] or coinfection with other pathogens, modulate the association between epilepsy and onchocerciasis.

The assessment of epilepsy prevalence in the various studies of the present analysis may have been influenced by the occurrence of other endemic diseases known to be involved in the aetiology of epilepsy [30]. Supposing a homogeneous distribution in the area of such a competing aetiological factor, the increase of epilepsy prevalence would be expected to comprise areas of low and high onchocerciasis prevalence to the same extent but would not affect an existing true association between epilepsy and onchocerciasis. In contrast, a co-endemic disease following a local distribution similar to that of onchocerciasis could reinforce (or weaken) this association. Neurocysticercosis caused by cerebral cysts of the pork tapeworm (*Taenia solium*) is considered a frequent cause for epilepsy in many sub-Saharan regions [30],[31] and it has been suggested that, in areas co-endemic for onchocerciasis and cysticercosis, epilepsy patients could be mistaken as having onchocerciasis-related epilepsy when they may be suffering from neurocysticercosis-derived epilepsy [32]. As far as information is available for the sites concerned in our analysis, cysticercosis was found endemic in the study areas in Burundi and Cameroon [7],[8],[33], but case-control studies from both these sites found a positive correlation between epilepsy and onchocerciasis. In one study from Uganda, serologic tests produced no evidence of a significant infestation of the population with *T. solium* [6]. In view of the results of the present analysis, we do not consider that neurocysticercosis could be a confounding factor that would produce a significant increase in epilepsy prevalence with increasing onchocerciasis prevalence. For this to be the case, it would be required that the distribution of neurocysticercosis (and thus the pig raising habits of the local communities) would closely follow the prevalence of onchocerciasis in the study areas of all seven countries involved.

An extensive literature comparing the performance of the various diagnostic methods of *O. volvulus* infection allowed us to develop appropriate correction factors for onchocerciasis prevalence. However, no such information is available for epilepsy that would have enabled us to do so for epilepsy prevalence. Strategies to assess prevalence of epilepsy in developing countries were developed in the 1980s [22],[30],[34],[35]. Ideally, such studies should follow a two-step protocol beginning with a population-wide door-to-door survey in order to (i) provide a complete census defining the sampling frame, (ii) assess epidemiologically relevant characteristics of the population and (iii) identify patients with possible epilepsy by use of a sensitive screening questionnaire. In a second step, patients with possible epilepsy are then subjected to a neurological and medical examination allowing confirmation or rejection of the diagnosis. Only two studies included in the present analysis can be regarded as having followed the recommended protocol [6],[18], but even these did not meet all requirements in that they did not use a pre-tested sensitive screening tool for patient identification but relied on a single screening question. This may have led to an underestimation of epilepsy prevalence in these studies. Procedures used for testing the population and identifying possible epilepsy patients, and for confirming diagnosis varied across the other studies and in a few instances these were not clearly specified (Table 2). Comparison between studies was also made difficult because no uniform definition for epilepsy was used. The relative weakness in neurological methods can in part be explained by the fact that most studies were initiated by researchers involved in onchocerciasis control measures and by the general scarcity of neurological expertise available in the endemic areas [34],[36].

The diversity of neuro-epidemiological methods may have influenced epilepsy prevalence obtained in the different studies. For instance, the use of community key informants for case identification may have resulted in an underestimation [37], whereas the lack of a confirmatory examination would have given a falsely high prevalence. Although we have to consider these effects as substantial, we were unable to express them quantitatively and our attempts to adjust epilepsy prevalence data were restricted to the study of Kipp et al. [19]. However, despite disparate neuro-epidemiological methods in use, we found epilepsy prevalence closely related to onchocerciasis prevalence throughout different African regions. It may be expected that this finding would be even more significant if methods with better accuracy were applied.

This is the first time that the relationship between the prevalences of onchocerciasis and epilepsy has been quantitatively assessed using available data collected at community level to perform adequate statistical analysis. We found that in areas where onchocerciasis is endemic, epilepsy prevalence increases with onchocerciasis prevalence. This is in accordance with the results of case-control studies and supports earlier anecdotal reports of numerous researchers working in various endemic areas [38],[39]. Because onchocerciasis is a disease affecting remote areas of countries with limited resources for neurological research, some of the studies carried out so far are influenced by methodological shortcomings. Future studies should use established and comparable protocols for neurological research. Possible research questions to be addressed with available and appropriate methods are: (i) the confirmation of the hitherto existing findings in other endemic areas; (ii) the role of intensity of infection in inducing epilepsy in areas not yet exposed to antifilarial treatment; (iii) the clinical characterization and classification of epilepsy in the various endemic areas; and (iv) the effect of long term onchocerciasis control on epilepsy incidence and prevalence.

## Supporting Information

Text S1 As the methods used to diagnose *Onchocerca volvulus* infection varied between studies, the prevalences of onchocerciasis were standardized using specifically developed tools. The details of the standardization methods are presented in this appendix.
(0.14 MB DOC)

Figure S1 Relationship between arcsine transformed onchocerciasis prevalence in the general (≥5 y.o. age group) population and onchocerciasis prevalence in 10–19 y.o. subjects across 51 villages located in the Mbam valley and continuous Lekie area (Cameroon) [2],[3],[4]. The line represents linear regression estimates.
(0.46 MB TIF)

Figure S2 *O. volvulus* microfilaridermia prevalence in communities from the Vina valley (North Cameroon) vs. onchocerciasis prevalence assessed from nodule palpation in males aged 20 y.o. and above [16]. Error bars represent 95% exact confidence limits of mean prevalence.
(0.49 MB TIF)

Figure S3 Evolution of onchocerciasis prevalence with annual ivermectin community treatments estimated through non linear regression (P_n_ = P_0_×q^n^) where P_n_ is the prevalence after n annual treatments, P_0_ is the initial prevalence and 1-q is the annual relative decrease assumed to be constant over time (q estimated as 0.926 [95%CI: 0.909–0.943]). The lines join the observations of a same study, without any assumption on the mathematical pattern of the decrease.
(0.42 MB TIF)
