# Supplementary material for: Epilepsy in Onchocerciasis Endemic Areas: Systematic Review and Meta-analysis of Population-Based Surveys
Source: PLoS Negl Trop Dis. 2009 Jun 16;3(6):e461. doi: 10.1371/journal.pntd.0000461 (PMC2691484; doi:10.1371/journal.pntd.0000461)
Supplement: Text S1 — As the methods used to diagnose Onchocerca volvulus infection varied between studies, the prevalences of onchocerciasis were standardized using specifically developed tools. The details of the standardization methods are presented in this appendix. (0.14 MB DOC) [file pntd.0000461.s001.doc]

**Supporting information to article:**

Epilepsy in onchocerciasis endemic areas: systematic review and meta-analysis of population-based surveys

**by** Pion, Kaiser, Boutros-Toni, Cournil, Taylor, Meredith, Stufe, Bertocchi, Kipp, Preux, Boussinesq

**Standardization of onchocerciasis prevalence**

The prevalence of onchocerciasis was considered standard if it has been estimated in the general population (≥ 5 years old (y.o.)) using the onchocerciasis diagnostic method used by the Onchocerciasis Control Programme in West Africa (OCP): this entails taking a skin biopsy from each iliac crest, using a 2 mm Holth-type punch, and incubating it for 24 h in normal saline before searching for the presence of *O. volvulus* microfilariae (mfs). All departures encountered in the different studies are listed in Table 1 of the article and specifically developed standardization procedures are presented below.

*Standardization of prevalence on the age-group examined*

Kaiser et al. [1] restricted their parasitological survey to individuals aged 10-23 years old with a time of residence in the study area between 10-19 years. To adjust for this recruitment bias, we characterized the relationship between the prevalence in the general population (≥ 5 y.o. age group) and the prevalence in the 10-19 y.o. age group. To do so, we analyzed results of parasitological surveys performed in 51 villages located in an onchocerciasis focus of central Cameroon where geographical characteristics appeared close to that in the Ugandan site [2,3,4] covering a total of 8911 individuals.

Linear regression analysis of community prevalence on prevalence in the 10-19 y.o. individuals was performed. However, since standard linear regression analysis involving variables that are proportions in not appropriate (the random variation is expected to be binomial rather than normal), arcsine transformation was applied [5]. The following relationship was estimated *y* = 56.45 *x* + 20.23 (R²=0.91) with *y*, the arcsine transformed prevalence in the general population and *x* = P_10-19_, the prevalence in the 10-19 y.o. age group (Figure S1). This relationship was used to adjust the prevalence values given by Kaiser et al. [1].

*Standardization of prevalence according to the diagnostic method*

(i) Consideration of differences in body sites at which the biopsies were taken

Gbenou [6] assessed the onchocerciasis prevalence from skin snips taken either at the scapula or at the iliac crests. We assumed that half of the patients included in this study had samples taken from the iliac crests, and then that the prevalence had to be corrected for the other half of the subjects. Onchocerciasis prevalence obtained from snips taken from the scapula or the iliac crests of the same individuals have been compared [7,8,9,10]. From these observations, we estimated the following linear regression model (using arcsine transformation for statistical reasons as above): *y* = 37.89 *x* + 35.94 (R²=0.50) where *y* stands for the arcsine transformed prevalence resulting from snips taken from the iliac crests and *x* for the prevalence resulting from snips taken from the scapula. This equation was applied to adjust the prevalences reported by Gbenou [6].

Taylor et al. [11] assessed onchocerciasis prevalence from skin snips taken from the lower anterior portion of one leg. These authors used sterile scalpel blades and lancets to excise a 5-7mm section of superficial skin, which is 2 to 3 times larger than skin snips obtained with a corneoscleral punch. To our knowledge, the relative sensitivity of skin snipping at this anatomic location has not been evaluated in comparison to the sensitivity obtained at the iliac crests (standard method). However, Barbiero and Trpis [7] and Ufomadu et al. [10] compared prevalence obtained from the iliac crests to prevalence obtained from the calf. The ratio between prevalence at the iliac crest and prevalence at the calf was very similar in the two studies (1.18 in the former study and 1.21 in the latter). However, we assumed that the large size of skin snips taken at the lower anterior portion of the leg compensated for the lower sensitivity at this location and we did not correct the prevalence found by Taylor et al. [11] in this respect .

(ii) Consideration of differences in incubation time of skin snips

In the study by Gbenou [6], the skin snips were incubated for 30 min in saline solution. The ratio between the prevalence obtained from skin snips incubated for 30 min and the prevalence from the same samples incubated for 24 h was assessed by Prost [12]. This author provides the evolution of prevalence from 30 min to 24 h in 16 villages whose onchocerciasis prevalence (at 30 min) ranged from 3.7% to 75.9%. The difference between the prevalence at 24h and the prevalence at 30 min ranged from 0 to 9.2% (mean difference (SD) = 3.5 (2.7)) irrespectively of the prevalence rate at 30 min. The mean value (3.5%) was added as a correction factor to the prevalence values reported by Gbenou [6].

Taylor et al. [11] searched for mfs after approximately 20 min of incubation. Given the rather large size (5-7 mm) of the skin samples collected as part of this study, it is reasonable to assume that, for a qualitative diagnostic test (presence versus absence of microfilariae), the sensitivity of a large skin sample incubated for 20 min is at least as good as the sensitivity of standard skin snips examined after 30 min. As for the Gbenou [6] data set, we added 3.5% to the prevalence rates found by Taylor et al. [11] to obtain the prevalence that would have been obtained at 24h.

(iii) Consideration of difference in sensitivity between skin snips and scarification methods

Newell et al. [13] used the scarification method to assess onchocerciasis prevalence in a hyper-endemic zone of Burundi. This method consists in performing a series of four 8-mm superficial incisions of the skin with a razor blade. The arising dermal juice is then collected and spread on a slide. After drying and staining with Giemsa stain, the microfilariae are identified and numbered using optical microscopy. The same author [14] compared the different diagnostic methods and concluded that the sensitivity of the scarification method was possibly slightly better than the skin snip method. Should a slight bias be introduced by the use of the scarification method, it would affect a single data point in a total of 91 communities. We decided not to adjust the prevalence values reported in the Burundi survey.

(iv) Estimation of microfilarial prevalence from nodule prevalence

In the study conducted in Central African Republic [15], onchocerciasis endemicity was assessed by the prevalence of onchocercal nodules in a group of male subjects aged 20 years and above. In order to correlate the prevalence of onchocercal nodules with the prevalence of microfilaridermia in this area, we analyzed data collected in a contiguous zone, the Vina valley (North Cameroon) [16]. The relationship between the prevalence of onchocerciasis estimated by assessing microfilaria in the skin and that estimated by nodule palpation can be described using the following logarithmic function: *y* = 28.5 Ln(*x*) + 73.95 (R²=0.67) with *y* being the arcsine transformed prevalence of microfilaridermia and *x* the prevalence of nodules (Figure S2). We used this equation to estimate the prevalence of microfilaridermia in the 42 villages surveyed by Druet-Cabanac et al. [15].

(v) Estimation of initial prevalence in areas with a history of large scale ivermectin treatment

This concerns three studies. The first one is the study by Dozie et al. [17] for which annual ivermectin treatments started in 1994 while the parasitological surveys were performed between March 1997 and December 2000. Therefore, 5 to 9 rounds of annual treatment were carried out before the parasitological surveys. Since no detail could be obtained on the actual number of annual treatments in each community, we used an assumed number of 7 for all of them.

The second study is by Kaiser et al. [1] for which 3 rounds of annual treatments had been carried out before the survey. The third is the study by Kipp et al. [18], performed in 1993 in an area where annual ivermectin distribution started in late 1991. We assumed that two annual rounds of ivermectin treatment had been conducted in this region before the onchocerciasis survey.

To estimate the initial prevalence in these areas, we developed a model estimating the progressive decrease in prevalence following repeated annual community treatments. This was done using published studies presenting the impact of community treatment with ivermectin on the prevalence of *O. volvulus* microfilaridermia [19,20,21,22,23,24,25]. The rationale behind this approach is that knowing the prevalence after a given number of treatments, it is possible to estimate the initial prevalence by using the reciprocal of the decrease function. We analyzed the results from studies reporting the effect of annual treatments on onchocerciasis prevalence. A non-linear regression was performed using the model P_n_= P_0_ x *q^n^* , where P_n_ is the prevalence after *n* annual treatments, P_0_ is the initial prevalence and 1-*q* is the annual relative decrease assumed to be constant over time during the first years [26]. Parameter *q* was estimated as 0.926 (95%CI: 0.909 – 0.943), R²=0.723, indicating that prevalence decreases approximately by 7% with each annual treatment (Figure S3). The observed values were thus corrected using the following relationship P_0_ = P_n_ x 0.926^-n^.

**References**

1. Kaiser C, Kipp W, Asaba G, Mugisa C, Kabagambe G, et al. (1996) The prevalence of epilepsy follows the distribution of onchocerciasis in a west Ugandan focus. Bull World Health Organ 74: 361-367.

2. Boussinesq M, Demanga-Ngangue, Richard P, Lele D, Cot S, et al. (1992) Etude clinique et parasitologique de l'onchocercose dans huit villages de la vallée du Mbam (Province du Centre, Cameroun). Bull liais doc - OCEAC 100: 26-31.

3. Macé J-M, Boussinesq M, Ngoumou P, Enyegue Oye J, Koeranga A, et al. (1997) Country-wide rapid epidemiological mapping of onchocerciasis (REMO) in Cameroon. Ann Trop Med Parasitol 91: 379-391.

4. Pion SDS (2004) Contribution à la modélisation des filarioses à *Onchocerca volvulus* et à *Loa loa* en Afrique centrale [PhD Thesis]. Créteil: Université Paris 12. 374 p.

5. Snedecor G, Cochrane W (1980). Statistical Methods. 7th ed: Iowa State University Press. pp. 290-291.

6. Gbenou HD (1995) Contribution à l'étude de l'association onchocercose-épilepsie. Résultats préliminaires d'une enquête neuroépidémiologique à Agbogbomé commune de Paouignan, sous-préfecture de Dassa-Zoumé, au Bénin [MD Thesis]. Cotonou: Université nationale du Bénin. 126 p.

7. Barbiero VK, Trpis M (1984) The prevalence of onchocerciasis on selected divisions of the Firestone Rubber Plantation, Harbel, Liberia. Am J Trop Med Hyg 33: 403-409.

8. Gbakima AA, Barbe RF (1992) *Onchocerca volvulus* infection in Sierra Leone: relation between prevalence, intensity of infection, and ocular problems in a 'forest' region. Acta Leiden 60: 47-59.

9. Mackenzie CD, Williams JF, O'Day J, Ghalal I, Flockhart HA, et al. (1987) Onchocerciasis in southwestern Sudan: parasitological and clinical characteristics. Am J Trop Med Hyg 36: 371-382.

10. Ufomadu GO, Eno RO, Akoh JI, Takahashi H, Uchida A, et al. (1988) Evaluation of skin biopsies from different body regions of onchocerciasis patients in Central Nigeria. Acta Trop 45: 257-262.

11. Taylor MM, Meredith SEO, Stufe A (1999) The prevalence of epilepsy in an area hyperendemic for onchocerciasis in Tanzania. American Journal of Tropical Medicine and Hygiene 61: 321.

12. Prost A (1976) La détection des snips faussement négatifs par lecture directe après une immersion de 24 heures en sérum physiologique. World Health Organization.

13. Newell ED, Vyungimana F, Bradley JE (1997) Epilepsy, retarded growth and onchocerciasis, in two areas of different endemicity of onchocerciasis in Burundi. Trans R Soc Trop Med Hyg 91: 525-527.

14. Newell ED (1997) Comparison of the use of skin scarification and skin biopsies to determine the prevalence and intensity of Onchocerca volvulus infection. Ann Trop Med Parasitol 91: 633-642.

15. Druet-Cabanac M, Bertocchi I, Preux PM (1998) Onchocerciasis and epilepsy in the North West region of Central African Republic. African Journal of Neurological Sciences 17: 37-38.

16. Remme JHF (2004) The global burden of onchocerciasis in 1990. Global Burden of Disease 1990. Geneva: World Health Organization.

17. Dozie IN, Onwuliri CO, Nwoke BE, Chukwuocha UM, Chikwendu CI, et al. (2006) Onchocerciasis and epilepsy in parts of the Imo river basin, Nigeria: a preliminary report. Public Health 120: 448-450.

18. Kipp W, Kasoro S, Burnham G (1994) Onchocerciasis and epilepsy in Uganda. Lancet 343: 183-184.

19. Boussinesq M, Chippaux JP, Ernould JC, Prod'Hon J, Quillevere D (1993) Efficacité parasitologique de traitements répétés par l'ivermectine dans un foyer d'onchocercose du Nord-Cameroun. Bull Soc Pathol Exot 86: 112-115.

20. Kennedy MH, Bertocchi I, Hopkins AD, Meredith SE (2002) The effect of 5 years of annual treatment with ivermectin (Mectizan) on the prevalence and morbidity of onchocerciasis in the village of Gami in the Central African Republic. Ann Trop Med Parasitol 96: 297-307.

21. Mas J, Ascaso C, Escaramis G, Abellana R, Duran E, et al. (2006) Reduction in the prevalence and intensity of infection in Onchocerca volvulus microfilariae according to ethnicity and community after 8 years of ivermectin treatment on the island of Bioko, Equatorial Guinea. Trop Med Int Health 11: 1082-1091.

22. Moyou-Somo R, Ngosso A, Dinga JS, Enyong PA, Fobi G (1993) A community-based trial of ivermectin for onchocerciasis control in the forest of southwestern Cameroon: clinical and parasitologic findings after three treatments. Am J Trop Med Hyg 48: 9-13.

23. Pacque M, Greene BM, Munoz B, Taylor HR (1991) Ivermectin therapy: a 5-year follow-up. J Infect Dis 164: 1035-1036.

24. Soumbey-Alley E, Plaisier AP, Boatin BA, Dadzie KY, Remme J, et al. (1994) The impact of five years of annual ivermectin treatment on skin microfilarial loads in the onchocerciasis focus of Asubende, Ghana. Trans R Soc Trop Med Hyg 88: 581-584.

25. Vyungimana F, Newell E (1998) Treatment of onchocerciasis with ivermectin (Province of Bururi, Burundi): parasitologic and clinical evaluation of different periodicities of treatment. Am J Trop Med Hyg 59: 828-831.

26. Winnen M, Plaisier AP, Alley ES, Nagelkerke NJ, van Oortmarssen G, et al. (2002) Can ivermectin mass treatments eliminate onchocerciasis in Africa? Bull World Health Organ 80: 384-391.
