# Supplementary figures and images for: Epilepsy in Onchocerciasis Endemic Areas: Systematic Review and Meta-analysis of Population-Based Surveys
Source: PLoS Negl Trop Dis. 2009 Jun 16;3(6):e461. doi: 10.1371/journal.pntd.0000461 (PMC2691484; doi:10.1371/journal.pntd.0000461)

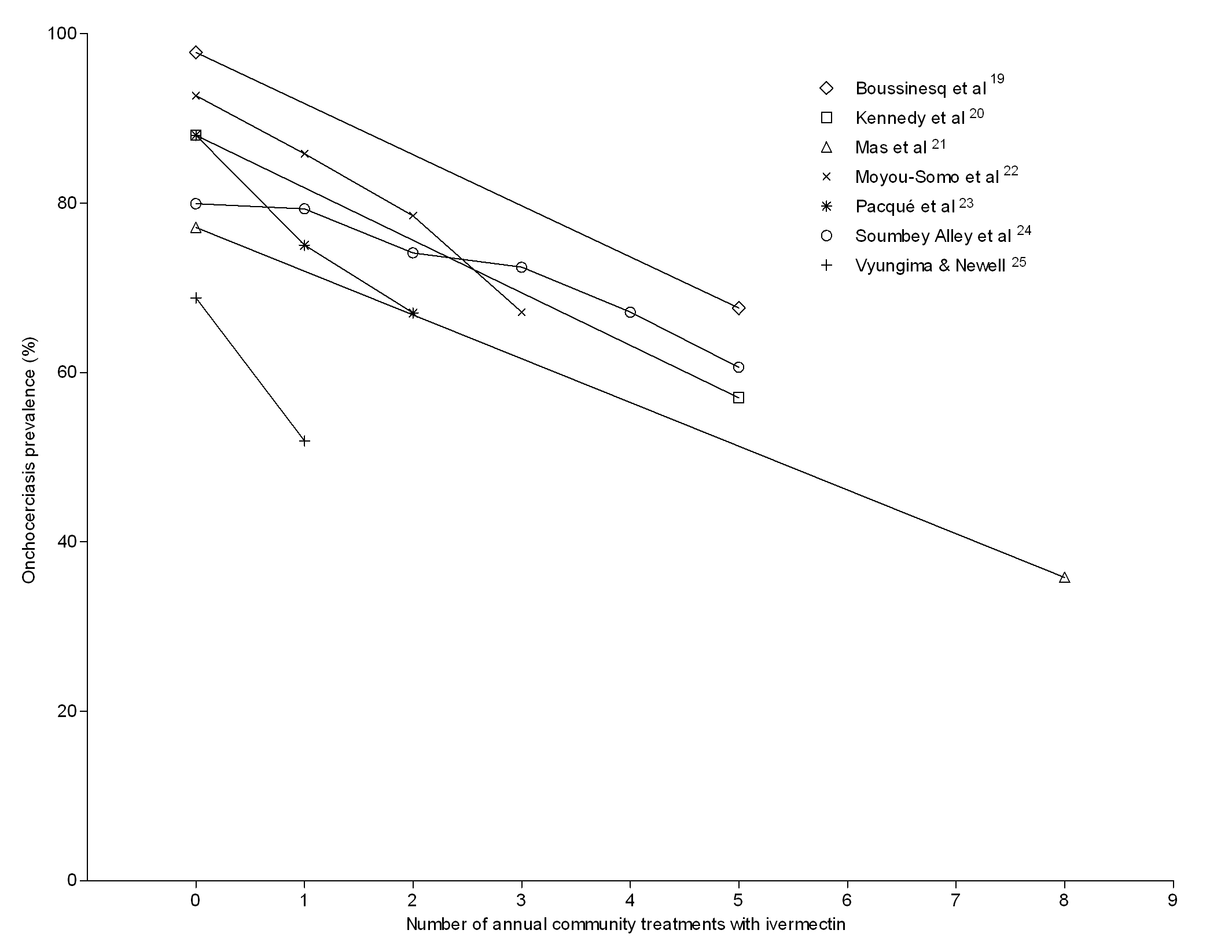

Supplement: Figure S3 — Evolution of onchocerciasis prevalence with annual ivermectin community treatments estimated through non linear regression (Pn = P0×qn) where Pn is the prevalence after n annual treatments, P0 is the initial prevalence and 1-q is the annual relative decrease assumed to be constant over time (q estimated as 0.926 [95%CI: 0.909–0.943]). The lines join the observations of a same study, without any assumption on the mathematical pattern of the decrease. (0.42 MB TIF) [file pntd.0000461.s004.tif]
